# Evaluability assessment as a design tool for complex health interventions: Insights from an EU-project on vaccine hesitancy

**DOI:** 10.17269/s41997-026-01148-3

**Published:** 2026-02-09

**Authors:** André Beja, Rita Morais, Cátia Sá Guerreiro, Tiago Correia

**Affiliations:** 1 Global Health and Tropical Medicine, GHTM, Associate Laboratory in Translation and Innovation Towards Global Health, LA‐REAL, Instituto de Higiene e Medicina Tropical, IHMT, Universidade Nova de Lisboa, UNL, Lisbon, Portugal; 2 WHO Collaborating Center on Health Workforce Policies and Planning, Instituto de Higiene e Medicina Tropical, Universidade Nova de Lisboa, Lisbon, Portugal

**Keywords:** Evaluability assessment, Evaluation, Health interventions, Vaccine hesitancy, Examen de l'évaluabilité, Évaluation, Interventions sanitaires, Hésitation vaccinale

## Abstract

**Background:**

Evaluability assessment (EA), conducted independently or as part of a broader evaluation process, is performed to determine whether a program, policy, or intervention is ready and suitable for evaluation. This study explores the innovative application of EA as a design-stage tool for guiding complex health interventions. It focuses on the VAX-TRUST *- Addressing Vaccine Hesitancy in Europe* project, where EA was employed to design and refine interventions addressing vaccine hesitancy across seven European regions.

**Methods:**

EA was applied in a qualitative approach using an *evaluability checklist*. This tool facilitated iterative improvements in intervention design, implementation planning, and evaluation readiness, ensuring interventions were robust and contextually relevant. Additionally, efforts were made to qualitatively measure the impact of the evaluability assessment through an *evaluability impact matrix*.

**Results and discussion:**

EA significantly influenced intervention design by identifying gaps and guiding real-time adjustments. Six of the seven interventions were successfully implemented with enhanced planning for unforeseen events, showcasing the iterative benefits of EA in the design phase. The iterative nature of EA allowed for real-time adjustments that enhanced program robustness and adaptability. Insights into the checklist’s gaps, such as underdeveloped resource allocation metrics, are discussed to improve future applications.

**Conclusions:**

This work advances EA literature by demonstrating its utility in the design stage and, by reinforcing the early alignment of different components and multiple contexts, provides a replicable framework for planning complex health interventions. Future evaluators are encouraged to adopt this proactive approach for more effective public health strategies.

## Résumé

**Introduction:**

Un examen de l'évaluabilité (EE) menée de manière indépendante ou dans le cadre d'un processus d'évaluation plus large, est réalisée afin de déterminer si un programme, une politique ou une intervention est prêt et adapté à l'évaluation. Cette étude explore l'application innovante de l'évaluation de l'évaluabilité en tant qu'outil de conception pour guider les interventions complexes dans le domaine de la santé. Elle se concentre sur le projet VAX-TRUST *- Addressing vaccine hesitancy in Europe*, dans le cadre duquel l'évaluation environnementale a été utilisée pour concevoir et affiner des interventions visant à lutter contre l'hésitation vaccinale dans sept régions européennes.

**Méthodologie:**

L'EE a été appliqué dans le cadre d'une approche qualitative utilisant une *liste de contrôle de l'évaluabilité*. Cet outil a permis d'apporter des améliorations itératives à la conception de l'intervention, à la planification de la mise en œuvre et à la préparation de l'évaluation, en veillant à ce que l'intervention soit solide et adaptée au contexte. En outre, des efforts ont été déployés pour mesurer qualitativement l'impact de l'évaluation de l'évaluabilité au moyen d'une *matrice d'impact de l'évaluabilité*.

**Résultats et discussion:**

L'EE a considérablement influencé la conception de l'intervention en identifiant les lacunes et en guidant les ajustements en temps réel. Six des sept interventions ont été mises en œuvre avec succès grâce à une meilleure planification des événements imprévus, ce qui illustre les avantages itératifs de l'évaluation environnementale au cours de la phase de conception. La nature itérative de l'EE a permis des ajustements en temps réel qui ont renforcé la robustesse et l'adaptabilité du programme. Les lacunes de la liste de contrôle, telles que les paramètres d'allocation des ressources sous-développés, sont discutées afin d'améliorer les applications futures.

**Conclusions:**

Ce travail fait progresser la littérature sur l'EE en démontrant son utilité au stade de la conception et, en renforçant l'alignement précoce des différents composants et des multiples contextes, fournit un cadre reproductible pour planifier les interventions complexes dans le domaine de la santé. Les futurs évaluateurs sont encouragés à adopter cette approche proactive pour des stratégies de santé publique plus efficaces.

## Introduction

Evaluability assessment (EA) studies evaluate the reliability and credibility of activities or projects (DAC-OCDE: Development Assistance Committee of the Organization for Economic Cooperation & Development, 2010). While traditionally employed to evaluate readiness or completed programs (Brunner et al., 2019), this study adopts a novel approach by integrating EA into the design phase of complex health interventions. This proactive use of EA seeks to ensure that programs are not only evaluable but are also designed with clarity, coherence, and adaptability from the outset, which ultimately enhances the evaluation process by strengthening the conditions for the implementation of all planned activities.

Evaluability studies originated in the late 1970's and have since become a valid and widely recognized tool for evaluators and program managers. This practice has been applied across various disciplines, including public health (Trevisan, 2007; Trevisan & Walser, 2015). Whether conducted in isolation or as part of broader evaluation processes, evaluability has been considered for multiple purposes, such as identifying promising interventions for evaluation, determining a program’s or intervention’s readiness for evaluation, supporting program development, and contributing to evaluation planning (Davies, 2013; Lam & Skinner, 2021; Trevisan & Walser, 2015).

Over time, the concept of evaluability has evolved and gained prominence, accompanied by a diversification of associated methodologies. However, the literature suggests that despite the development of various models for conducting evaluability studies, no single approach has emerged as a definitive standard.

Vaccine hesitancy means a delay in acceptance or even refusal of vaccines, despite their availability and the accessibility of vaccination services, and it is seen as a highly complex and contextual phenomenon that varies by time, location, and even the specific vaccines in question (SAGE: Strategic Advisory Group of Experts on Immunization, 2014).

There is ongoing debate regarding the conflation of vaccine hesitancy with vaccination status. While low vaccine uptake is often used as a proxy for hesitancy, these are distinct phenomena: individuals may remain unvaccinated due to structural or access-related barriers rather than attitudinal factors, whereas others may be hesitant yet vaccinated because of mandates or social pressures (Bedford et al., 2018). Misinterpreting coverage data as direct evidence of hesitancy risks driving inappropriate interventions. To avoid this, our study adopted the concept of hesitancy, with interventions explicitly designed to address attitudinal and relational determinants. This distinction informed both the evaluability assessment and the development of region-specific interventions, ensuring they were responsive to the underlying drivers of hesitancy, rather than merely seeking to increase vaccination rates.

Therefore, vaccine hesitancy should be viewed as a multifaceted phenomenon posing threats to global health (WHO, 2019) posing challenges that require tailored interventions (Collins, 2014; Yaqub et al., 2014). Depending on their scope and attributes, these interventions can take the form of complex health interventions, characterized by involving multiple interactive components, targeting various behaviours, and requiring adaptation and flexibility to different groups, contexts, and delivery settings, often requiring specialized knowledge on the part of those providing and receiving the intervention (Skivington et al., 2021).

Addressing vaccine hesitancy demands contextually appropriate strategies that balance theoretical frameworks with practical considerations. This study explores the innovative application of evaluability assessment (EA) as a design-stage tool for guiding complex health interventions, in this case by guiding the design of interventions that address vaccine hesitancy effectively across diverse contexts. This novel application addresses a key gap in the literature by demonstrating the proactive utility of EA in designing interventions that are adaptable and aligned with diverse regional contexts.

To develop recommendations for international, national, and local public health authorities to support health professionals in understanding and addressing vaccine hesitancy, the EU-funded VAX-TRUST - *Addressing Vaccine Hesitancy in Europe* project involved ten partners from seven countries -Finland, Belgium, Poland, Italy, Portugal, the Czech Republic, and the United Kingdom (UK)- in an action-research initiative. This initiative comprised (a) gathering evidence for a situational analysis of vaccine hesitancy in Europe, with emphasis on the regions where the project took place; (b) conducting ethnographic research to gain new perspectives on vaccine encounters; and (c) designing and implementing evidence-based and context-focused interventions to raise awareness and prepare health professionals to engage with hesitant individuals (Vuolanto et al., 2024).

Guided by a common framework adopted by the project partners, these complex health interventions were implemented across seven European regions (Helsinki region, Flanders, Piemonte, Małopolska, Prague region, Lisbon region, East Midlands) by local project partners, features that give them a complex character, and underwent a comprehensive evaluation process conducted by a team specifically assigned to this task, which supervised the design of each intervention and then monitored its implementation in the field. This evaluation process included an evaluability assessment and implementation analysis, aimed at reinforcing the initiatives’ underlying assumptions and execution while validating the results achieved (Davidson et al., 2019; Sá Guerreiro et al., 2022; Skivington et al., 2021).

To explore broader uses of evaluability in complex health interventions, particularly those related to vaccine hesitancy, this article describes and discusses the results of the evaluability analysis that focused on the general framework for designing the VAX-TRUST project health interventions and on the intervention plans that, framed by this guideline, were developed by the partners in each of the seven target regions where the project took place.

The expected contribution to the debate involves providing insights into the effectiveness of evaluability studies for complex health interventions, highlighting the diversity in methodologies, and emphasizing the need for standardized models. This is particularly relevant to inform and support public health authorities and professionals in better understanding and managing vaccine hesitancy.

## Methods

This study utilized EA as a proactive tool during the interventions design stage, diverging from traditional applications that typically assess readiness post-design. It was conducted by a team of researchers based in Lisbon, independent from the other teams in the consortium and with backgrounds in public health and vaccination, as well as in intervention planning and evaluation.

Recognizing that evaluability frameworks are often rooted in Western traditions of rational planning and linear causality, steps were taken to mitigate potential biases during the EA process. According to the project logic, the team that conducted the EA and, through it, supported planning, had no direct contact with stakeholders in each target region, as this was the responsibility of local partners. Furthermore, these partners were strongly encouraged to include stakeholders at various levels during the design and implementation of interventions, ensuring that local actors in each region had a voice in interpreting concepts such as coherence, feasibility, and relevance. Feedback loops were designed to surface context-specific knowledge, challenge assumptions embedded in dominant logic models, and adapt the checklist criteria to reflect regional realities. While the guiding framework was informed by international literature, its application was iterative and responsive, privileging local perspectives over prescriptive templates.

Methodologically, a qualitative approach was purposefully designed, adhering to established procedures for conducting evaluability studies as outlined in the literature (Davies, 2013; Davies & Payne, 2015; Trevisan & Walser, 2015). This approach was implemented in two phases:

- Phase 1: A detailed* evaluability checklist* was developed to analyse the general framework guiding intervention design. From the checklist, it was possible to get a comprehensive overview of this intervention planning model and assess its relevance, coherence, and resource adequacy, while identifying gaps that required resolution before implementation.
- Phase 2: The checklist was applied to seven region-specific intervention proposals, followed by iterative feedback loops to refine these plans in response to context-specific challenges.

The EA process incorporated researcher triangulation, stakeholder engagement (i.e., local planners, who, in turn, were encouraged to involve local players with a keen interest or potential to act on vaccine hesitancy), and iterative recommendations to address variability in regional contexts and align interventions with the project’s overall framework. Key challenges included balancing evaluability criteria with diverse local stakeholder priorities and addressing resource disparities across regions. These challenges were mitigated through tailored guidance, collaborative discussions, and alignment strategies. Furthermore, during the analysis of the implementation process of the interventions that followed their completion, the evaluators based in Lisbon made efforts to retrospectively understand the impact of EA on the design and implementation of each intervention.

### Data collection

An *evaluability checklist* (see Table 3 in the Annexes) was developed to address the evaluability objectives and challenges identified in the first phase. This checklist was applied to the VAX-TRUST project’s general framework of interventions to determine its suitability for designing evaluable interventions and to propose improvements for its final version. Subsequently, the checklist was used for similar purposes in analyzing the specific intervention proposals developed in each of the seven target regions. The checklist is grounded in the main checklists available in the literature, primarily from renowned international agencies (ILO, 2020; Evaluation Office, 2019; UNFPA Evaluation Office, 2019; UNDOC, 2017; Davies, 2013).

To qualitatively capture, summarize, and present the main results clearly, three key parameters were defined: (1) the relevance, logic, and coherence of the theoretical background and intervention design, and their alignment with the context of the target region; (2) the adequacy and validity of the indicators, tools, and systems for monitoring, measuring, and verifying the results; and (3) the adequacy of the financial, human, and material resources required to achieve the expected results and plan the evaluation.

Additionally, an *evaluability impact matrix* was developed to assess the impact of the evaluability assessment on the planning and implementation processes of each intervention, as well as on the achieved results (see Table 4 in the Annexes). This tool was structured based on the *evaluability checklist* and informed by the contributions of various authors (Bichelmeyer, 2003; Scriven, 2007; Stufflebeam, 2000a, 2000b). Outside the scope of the evaluability analysis carried out at the interventions planning stage, this matrix was applied to each of the seven interventions, populated with data extracted from the intervention plan, implementation report, and evaluation report produced by a local evaluator. This allowed for a comparison between what was planned and what was implemented, enabling an assessment of the impact of evaluability.

### Data analysis

In both EA phases, content analysis was applied to all available documents relevant to the evaluability study. The *data corpus* consisted of the VAX-TRUST project deliverables, as detailed in Table 1. As foreseen in content analysis, the documents were carefully reviewed to identify significant information relevant to the evaluability assessment. This significant information was used to complete the *evaluability checklist* and populate the *evaluability impact matrix*. Notably, the same data could be applied to more than one category of the checklist/matrix.

**Table 1:** Documents analysed during the study

Private documents
• Framework to design the VAX-TRUST project interventions (draft version) • Document containing a preliminary assessment of the proposed framework for designing interventions (email from the evaluation team to the partners) • Design proposals for interventions in the 7 target regions (draft version) • Document with a preliminary assessment of the proposed interventions in each target region (email from the evaluation team to the partners)
Public documents
• VAX-TRUST project Deliverables 5.1 and 5.2 (de Vito et al., 2022a, 2022b), containing the final version of: ○ the intervention design framework ○ plans of the interventions to be developed in each of the 7 target regions • VAX-TRUST project Deliverable 6.1, containing the evaluability assessment report to the intervention design framework (Sá Guerreiro et al., 2022) • VAX-TRUST project Deliverable 5.3, containing the seven implementation reports of the interventions (de Vito et al., 2023)—Drawn up after the implementation phase of the interventions, this report was intended to provide a retrospective understanding of the impact of evaluability • Evaluation reports on the interventions in each target region, drawn up by an external evaluator (can be found in Deliverable 6.3 of the project) (Sá Guerreiro et al., 2024)—Drawn up after the implementation phase of the interventions, this report was intended to provide a retrospective understanding of the impact of evaluability

Subsequently, the data were systematically and semantically reviewed and discussed by the authors to provide suggestions for improving the intervention design and, in a later moment, to assess the impact of evaluability on the implementation of the interventions. Researcher triangulation was employed to ensure the quality of the data analysis and its results. Any discrepancies in data interpretation were discussed to reach a consensus.

## Results

Given the aim of the article, the results are presented with a focus on three main aspects related to availability assessment and its results: First, we will explore the evaluability assessment of the general framework designed to guide intervention development in each target region, which is followed by an overview of the approach applied to the seven intervention proposals developed by various partners based on this framework. Furthermore, to elucidate the significance of those approaches, the impact of the recommendations generated through the evaluability assessment on the implementation of the interventions will be explored.

### Evaluability assessment of the general framework

The content analysis of the framework’s proposal revealed a model that aligns closely with the principles and objectives of the VAX-TRUST project. Grounded in sociological theory, which provides a comprehensive lens for understanding vaccine hesitancy, and supported by theories guiding the design of complex public health interventions, the framework establishes a robust foundation for developing interventions tailored to specific contexts and constraints of each target region. As a guiding tool, this framework holds significant value in facilitating the design of interventions that are both nuanced and context-specific while remaining aligned with shared objectives and possessing strong evaluative potential.

Nonetheless, a detailed examination of the proposal’s structure and content, guided by the *evaluability checklist*, revealed a lack of clarity in delineating the process for developing interventions, which could hinder the effective translation of theory into practice. This analysis highlighted the need for clearer conceptual elucidation and practical guidelines for their application. Additionally, it emphasized the importance of establishing feasible and measurable objectives and methodologies early in the planning process, alongside anticipating resource requirements and developing protocols for monitoring activities and outcomes.

These findings informed recommendations to enhance the framework, with the goal of streamlining the design and implementation processes for interventions across diverse regions and contexts, thereby facilitating their continuous and effective evaluation. The recommendations were discussed with the partners responsible for formulating the framework, and most were incorporated into the final version.

### The approach taken to the intervention proposals in the different target regions

The evaluation team monitored the planning processes in each TR, providing suggestions to improve and strengthen the intervention designs as needed. These efforts aimed to ensure that the designs were in optimal condition for implementation and evaluation. Subsequently, the plans underwent an evaluability assessment, which examined the assumptions and contents of each intervention proposal. This assessment also evaluated the internal coherence of the proposals with the design framework and their external alignment with the principles of the VAX-TRUST project.

Despite the varying degrees of maturity among the proposals, six of the seven interventions (I1, I2, I3, I4, I5, I6) were deemed evaluable and aligned with the project’s overall orientation and assumptions. Recommendations were tailored to the specific contexts of each region, aiming to help partners adapt to the project’s constraints and ensure the availability of evidence suitable for evaluation. These recommendations emphasized (i) justifying each proposal with an adequate theoretical background and a comprehensive contextual analysis; (ii) formulating realistic, measurable, and specific objectives; (iii) providing a detailed definition of the target population, including strategies for recruitment and participant retention; and (iv) describing the methods to be implemented and thoroughly planning the proposed activities, including the timelines and resource requirements.

The importance of articulating the expected short and medium-term results was also emphasized, along with the need to establish clear indicators and mechanisms to measure and report the intended results and operationalize the internal evaluation of each intervention. Finally, partners were advised to systematize a risk management and mitigation plan addressing operational, contextual, and evaluation-related risks. These included potential disruptions such as delays in recruiting target populations, turnover of key personnel, changes in local vaccination policies, or shifts in attitudes that could affect engagement. Importantly, risks also encompassed evaluation challenges, such as the possibility that iterative adaptations to the intervention might misalign with the monitoring framework or indicators established during planning. By anticipating these scenarios, local teams could define contingency measures to preserve data integrity and ensure that evaluation evidence remained robust despite unforeseen changes.

Regarding the seventh intervention (I7) plan, despite the merit of its contents and the efforts of the team that developed it, the plan exhibited a lower degree of alignment with the intervention framework. Key elements necessary to ensure its implementation within the project’s timeframe and assumptions, as well as its evaluability, were lacking. To address these issues, recommendations were provided to assist the partners in adapting their plan to the project’s constraints and ensuring the availability of evidence for evaluation.

The recommendations for the intervention drafts are summarized in Table 2, categorized by the dimensions addressed, along with a qualitative appraisal of the level of integration (i.e., accepted, partially accepted, or rejected) by the local partners in the final version of each plan.

**Table 2:** Recommendations made for each intervention plan during the evaluability assessment (number and level of integration in the final version)

Evaluability dimension	Recommendations (topic to complete, clarify or reinforce)	Intervention 1 (I1)	Interv.2 (I2)	Inter.3 (I3)	Interv.4 (I4)	Interv.5 (I5)	Interv.6 (I6)	Interv.7 (I7)
Relevance, logic, and coherence of the intervention theoretical background and design and their alignment to the target region context	Intervention overview				A (1)		A (1)	PA (1)
	Theoretical background	A (1)	A (1)	A (1)		A (1)		PA (1)
	Target region context	A (1)	A (1)	PA (1)	A (1)	A (1)	A (1)	PA (1)
	Objectives			PA (1)		A (1)		
Adequacy and validity of the indicators, tools, and systems for monitoring, measuring and verifying results	Methods, materials and programming	A (1) PA (1)	A (1) PA (2)	A (4)	A (2) PA 1	A (3)	A (2)	A (1) PA (2)
	Target population and participant recruitment	A (1) R (1)	A (1) R (1)	A (3)	A (1) PA (1)	A (2)	A (1) R (1)	PA (1) R (1)
	Monitoring, evaluation, and reporting	PA (1)	PA (1) R (2)	PA (1)	PA (2)	A (1)		R (1)
	Risks and mitigation plan	A (2)	A (1) PA (1)	PA (1)		A (1)	A (1)	PA (1)
Adequacy of financial, human, and material resources to meet the expected results and planning evaluation	Trainees’ profile and tasks		A (1)	A (1)	PA (1)	A (1)		
	Budget plan	A (1)					A (1)	R (1)

*N* number of evaluability recommendations, *A* accepted, *PA* partially accepted, *R* rejected

### Impact of the evaluability assessment on the implementation of interventions

Six interventions (I1–I6) were implemented and evaluated according to the final version of the plan. The evaluators’ implementation analysis suggests that the evaluability recommendations led to more detailed planning and a greater capacity to manage unforeseen events, contributing to more impactful results and more solid evaluations. It also suggests that some of the difficulties registered during the process may be due to aspects that, having been pointed out in the EA, were not properly considered by the partners in each TR.

Through the *evaluability impact matrix*, it was possible to gain a retrospective and more comprehensive understanding of how integrating evaluability recommendations into planning contributed to these outcomes. This meant reviewing each of the suggestions, even those not accepted by the planners in each TR, and checking how the topic it addresses was, or was not, referred to in the evaluators’ report. It was possible to identify topics that (i) were not mentioned by the evaluators, which can be interpreted as a reflection of the positive impact of the recommendation; (ii) were considered to be process or result enhancers; and (iii) were associated with some of the implementation difficulties because, despite the recommendations, they were not sufficiently developed or covered in the planning. Below are some examples to illustrate each of these situations.

The reinforcement of the theoretical and contextual assumptions regarding how to deal with vaccine hesitancy and the characterization of the specific context of TR, or the need to define a clearer risk mitigation measure within planning, among others, were topics addressed in five of the recommendations to I1 and mentioned in the evaluator report, not necessarily associated with positive or negative implementation and evaluation concerns. This shows the relevance of the issue and that the changes made have probably made it possible to avoid negative effects.

Of the 11 recommendations to strengthen the assumptions of I2, six were accepted, three were partially met, and two were not considered in the final version of the plan. Those topics that were not fully included were related to the suitability and validity of the indicators, tools, and systems for monitoring, measuring, and verifying results. As anticipated during the EA, the evaluators point to the lack of a clearer definition on central themes for the evaluation of this intervention (for example, indicators to determine the extent to which results have been achieved) as the cause of an additional difficulty in the implementation process and in the evaluation of the intervention itself.

Part of the recommendations made for I3 planning pointed to the need to improve the definition of objectives and, which would lead to greater clarity, and, as a continuation of this, to clarify the results to be achieved and the procedures relating to the internal monitoring and evaluation of the intervention. Despite the modifications made to the objectives, the evaluator still mentions difficulties in implementation that could have been mitigated if some of the suggestions made had been addressed more thoroughly.

No difficulties related to previously foreseen and uncorrected planning issues were clearly mentioned in the I4 evaluation report. However, carrying out internal and external evaluations could have been simplified if there had been a more detailed definition of how the results achieved should be measured and the baseline data with which they should be compared.

The EA allowed for changes to the I5 plan that gave a clearer and more focused view of implementation, results, and even internal evaluation. Examples of this are the improvements made to the definition of the target population and the measures to ensure their recruitment, as well as the introduction of greater clarity regarding the materials to be used and the profile and roles of the trainers.

Regarding I6, the need to improve the description of the vaccination context in the TR led to robust changes in the intervention plan. Even so, this topic is mentioned in the evaluators’ implementation report as one of the aspects that the participants would have liked to have seen more of during the discussion sessions. These two seemingly contradictory facts illustrate the challenges inherent in evaluability in this specific process: identify weaknesses that are recognized and accepted by those responsible for planning and that lead them to trigger concrete changes, not just formal statements, which have repercussions on the different levels.

The seventh (I7) encountered setbacks that affected its course and was not completed within the timeframe set in the project. The evaluator noted a series of difficulties that conditioned this process, some of them related to aspects that, having been pointed out in the EA, did not result in changes to the planning, a result that should be seen as learning about the need for a permanent and open dialogue between evaluators and planners as a way of ensuring the alignment and success of interventions.

## Discussion

Evaluability assessment demonstrates formative value when integrated into the design phase of complex interventions and, under certain conditions, may also contribute to transformational change by fostering alignment among stakeholders and improving intervention coherence. The literature highlights that EA can mitigate risks of null or negative outcomes in interventions by addressing factors such as incomplete or non-existent implementation, unrealistic goals, illogical design, or conflicting goals (Davies, 2013). Moreover, EA helps protect the evaluation process from being undermined by poor planning practices, including lack of independence in evaluation teams, insufficiently specific objectives, absence of clearly defined indicators, or missing baseline data for comparison (Ruben, 2012).

Many of these issues stem from the design process, underscoring the critical importance of evaluability at this stage. By identifying and addressing design gaps early, EA enabled the development of more coherent and evaluable interventions that were tailored to the specific contexts of each region.

The evaluability study within the VAX-TRUST project interventions specifically addressed these aspects, with a particular focus on the efficiency of the planning processes. This approach facilitated a more consistent design of the interventions and strengthened their implementation and evaluation processes, thereby contributing to the project’s objectives. Several lessons include the following:

- Iterative benefits: The iterative nature of EA strengthened planning processes by enabling adaptive adjustments to unforeseen challenges encountered during implementation.
- Gaps in EA tools: The *evaluability checklist* highlighted areas for improvement, particularly in providing guidance on tracking resource allocation and participant engagement metrics. Addressing these gaps in future iterations of EA tools could significantly enhance their utility and applicability.
- Stakeholder engagement: Active collaboration with stakeholders played a pivotal role in aligning interventions with both theoretical frameworks and practical realities. This engagement fostered a sense of ownership and enhanced adaptability across diverse regional contexts.

The methodology employed in this study was innovative, particularly in terms of the instruments developed for its specific purposes and adhered to the guidelines described in the literature. Notable similarities were observed with established proposals in this field, especially regarding the recommended steps for implementation: (1) identifying the project’s scope and expected outcomes of the evaluability assessment; (2) determining the resources available for analysis; (3) reviewing the available documentation; (4) engaging stakeholders; (5) developing recommendations; and (6) providing feedback to the stakeholders. Furthermore, the recommendations should address the project’s rationale and design, the monitoring and evaluation system and its capacity, evaluation issues relevant to stakeholders, and potential evaluation designs (Brunner et al., 2019; Davies, 2013; Lam & Skinner, 2021).

The evaluability checklist developed for this study provided comprehensive and visible coverage of the most critical issues related to complex interventions. As a tool not limited to a specific theme or intervention, it holds potential for broader application in analyzing other complex health interventions. This checklist is particularly noteworthy for enabling a straightforward and intuitive preliminary analysis, complemented by detailed comments for in-depth evaluation. Given the multidimensional, contextual, and time-sensitive nature of vaccine hesitancy, interventions addressing it must reflect these complexities. In this context, the checklist proved to be a robust and effective tool for designing and evaluating such interventions.

While the checklist proved valuable for strengthening the design and implementation of complex interventions, its utility may vary across different evaluation approaches. Goal-based and theory-driven evaluations particularly benefit from such structured criteria, as they require clear objectives, indicators, and resources to ensure coherence between design, implementation, and outcomes. Conversely, goal-free evaluation (GFE) approaches, where evaluators deliberately avoid knowledge of predefined goals or objectives, may be less dependent on these criteria (Youker & Ballard, 2024). By focusing on identifying actual rather than intended outcomes, GFE minimizes some risks associated with incomplete or poorly articulated designs and can uncover unintended effects or contextual changes that goal-based approaches might miss. Thus, while our checklist supports evaluability in most traditional evaluation contexts, it should be seen as complementary rather than universally applicable. In settings where GFE is appropriate, such as early exploratory phases or highly dynamic environments, evaluation efforts may proceed effectively without the extensive goal-orientation required by our framework.

Future applications of EA should prioritize sustained stakeholder collaboration, not only as a means of contextual adaptation but as a core evaluability criterion. Adequate resources and dedicated time must be allocated for engaging diverse stakeholders throughout the design, implementation, and evaluation phases. This includes supporting local actors’ capacity to interpret evaluability findings and co-create solutions, thereby strengthening the relevance and legitimacy of complex health interventions.

It is also important that future approaches anticipate and monitor potential unintended negative impacts of the EA process itself. These may include delays caused by iterative revisions, stakeholder fatigue from repeated consultation cycles, or disruptions to monitoring systems when design changes misalign with baseline indicators. By incorporating reflexive mechanisms, such as real-time feedback loops and explicit documentation of trade-offs, evaluators can balance the benefits of adaptive planning with the need to preserve evaluation integrity and avoid harm to program delivery.

Another key consideration for future applications of this *evaluability* *checklist* is its flexibility to address region-specific contexts. While the checklist was developed from international guidance and successfully applied across diverse European regions in this study, its utility and impact could be further enhanced through localized adaptations. This may include translating the tool into local languages, integrating the realities of specific health system structures, and aligning with community engagement practices that reflect cultural norms and expectations. Such tailoring would not alter the core evaluability principles but would ensure that the criteria resonate with local stakeholders and capture context-sensitive factors influencing intervention design and implementation. By embedding these regionally relevant adaptations, future EA processes can strengthen stakeholder ownership and improve the relevance and legitimacy of findings, ultimately contributing to more sustainable and effective public health interventions.

## Conclusion

In summary, with the WHO recognizing vaccine hesitancy as a major global health threat, interventions to address this issue must aim for maximum effectiveness. This study contributes to the evaluability assessment literature by demonstrating its utility as a design-stage tool for complex health interventions. Integrating EA into the early stages of program development ensures that interventions are both contextually relevant and evaluable, ultimately enhancing their overall effectiveness. The insights derived from this iterative process highlight the critical role of proactive evaluation in addressing complex public health challenges, such as vaccine hesitancy. Future applications of EA should prioritize stakeholder collaboration, adaptability, and iterative feedback to further refine intervention design and optimize outcomes.

## Contributions to knowledge

What does this study add to existing knowledge?

- This study introduces evaluability assessment (EA) as a design-stage tool for complex health interventions, shifting from its conventional retrospective use.
- It demonstrates how EA can enhance intervention feasibility, coherence, and adaptability from the outset, optimizing evaluation readiness. By applying EA in the VAX-TRUST project on vaccine hesitancy, it presents a novel, proactive approach to health evaluation, bridging the gap between design and assessment.
- This work offers a replicable framework that strengthens intervention planning and contributes to the evolution of evaluation methodologies in public health.

What are the key implications for public health interventions, practice, or policy?

- This study positions evaluability assessment as a proactive strategy in public health intervention design, ensuring feasibility before implementation.
- Embedding EA early allows policymakers to refine frameworks, optimize resources, and address challenges in advance.
- This approach shifts evaluation from reactive to iterative, making interventions more adaptive and evidence-based.
- The findings advocate for institutionalizing EA in health policy, strengthening public health strategies by ensuring interventions are not only theoretically sound but also practically viable.

## Data Availability

Five project deliverables/reports are pointed out in the “References” section. In accordance with the project’s grant agreement, these are open access materials. The authors of two of the reports cited are the same as those of the article. The authors of the other three project deliverables cited have been informed of the use of this material.

## Annexes

**Table 3:** The Evaluability Checklist

Evaluability Parameter/Dimension	Key Questions/Subdimensions	Short analysis	Recommendations
Assess the relevance, logic and coherence of the intervention theoretical background and design and their alignment to the target region context	• Does the intervention and its specificities have a clear theory of change/logic model?	*Have a clear theory of change/logic model *	
		*Have a theory of change/logic model needing explanation *	
		*Does not have a clear theory of change/logic model*	
	• Does it address the problem identified in VAX-TRUST PROJECT?	*The problem is fully addressed *	
		*The problem is addressed *	
		*The problem is not addressed *	
	• Is the design framework of the intervention coherently articulated and aligned to the VAX-TRUST PROJECT plan?	*Fully articulated and aligned *	
		*Articulated and aligned *	
		*Not articulated or aligned *	
	**• **Is it aligned to the target region context?	*Fully articulated and aligned *	
		*Articulated and aligned *	
		*Not articulated or aligned *	
		*Unknown or difficult to attend to all specificities of the different target regions *	
	• What is the context underlying the intervention?		
	• What is the logical framework underlying the intervention?		
	• What main goals are set?		
	• Are the objectives related to the goals?	*Totally related *	
		*Related *	
		*Unknown or not related *	
	• Who will be the recipients/beneficiaries of the intervention?		
	• What activities are planned?		
	• What results are expected in the short and medium term?		
Assess the adequacy andvalidity of the indicators, tools and systems formonitoring, measuringand verifying results	• Are the expected results clear, realistic and in line with the main goals and objectives?	*Totally clear, realistic, and aligned *	
		*Clear, realistic, and aligned*	
		*Unknown, or not clear, realistic or aligned*	
	• Are they measurable (quantitatively or qualitatively)?	*Totally measurable*	
		*Probably Measurable *	
		*Unknown or not measurable *	
	• Does the intervention have the capacity to provide data for monitoring and evaluation?	*Totally capable *	
		*Capable *	
		*Not capable *	
	• Is the way to evaluate the activities clearly defined?	*Clearly defined *	
		*Defined *	
		*Not clearly defined *	
	• Is the way to evaluate the results clearly defined?	*Clearly defined *	
		*Defined *	
		*Not applicable at this time or not clearly defined *	
	• To what extent are results and indicators measurable?	*Totally measurable *	
		*Measurable *	
		*Not measurable *	
	• Are baselines in place for evaluation indicators?	*There are baselines in place for evaluation indicators*	
		*There are no baselines in place for evaluation indicators*	
	• Does the intervention have a monitoring system to gather and systematize the information with defined responsibilities, sources and periodicity?	*There is a monitoring system*	
		*There is no monitoring system*	
	• Are there plausible plans to monitor these in any practicable way?	*There are plans to monitor the interventions in a practicable way *	
		*There are no plans to monitor the interventions in a practicable way*	
	• Have assumptions about the role of WP’s partners in the intervention designing been made explicit?	*Totally explicit*	
		*Explicit *	
		*Not explicit *	
	• How well have key assumptions, risks and mitigation strategies been specified?	*Totally specified *	
		*Specified *	
		*Not specified *	
	• Is there a viable plan to fill data gaps?	*There is a viable plan *	
		*There is no viable plan *	
Assess the adequacy of financial, human and material resources to meet the expected results and planning evaluation	• What resources will be involved?		
	• Are all the resources aligned with the results?	*Fully aligned *	
		*Aligned *	
		*Unknown or not aligned *	
	• Are data and systems in place to allow VAX-TRUST PROJECT to assess the adequacy of resources to achieve intended results?	*Data and systems are fully in place *	
		*Data and systems are in place*	
		*Data and systems are not in place *	
	• Are there resources set aside for the evaluation?	*Are resources set aside for the evaluation *	
		*There are no resources set aside for the evaluation *	
	• Is there adequate, monitoring, evaluation and learning in place for the intervention to be evaluated?	*Monitoring, evaluation, and learning are totally in place for the intervention evaluation *	
		*Monitoring, evaluation, and learning are in place for the intervention evaluation*	
		*Monitoring, evaluation, and learning are not in place for the intervention evaluation*	
	• Does the intervention include a clear plan and budget for evaluation?	*The plan and budged for evaluation are included*	
		*The plan and budged for evaluation are not included*	

**Table 4:** Evaluability Impact Matrix

TARGET REGION: (NAME)				
Evaluability Parameters/ dimensions	Evaluability Suggestions	Translation of the recommendation into the intervention plan	Evaluation report mentions the issue that led to the recommendation	Comment on impact of the recommendation (how the subject is reflected in the report)
Assess the relevance, logic and coherence of the intervention theoretical background and design and their alignment to the target region context	*Suggestion 1*	*Fully attended*	*Mentions the topic*	
		*Partially attended *	*Mentions the topic associating it positively to the implementation and/or results *	
		*Not attended *	*Mentions the topic associating it with implementation difficulties*	
		*Not applicable*	*Does not mention the topic*	
	*Suggestion 2*	*Fully attended*	*Mentions the topic*	
		*Partially attended *	*Mentions the topic associating it positively to the implementation and/or results *	
		*Not attended *	*Mentions the topic associating it with implementation difficulties*	
		*Not applicable*	*Does not mention the topic*	
	*Suggestion (…)*			
Assess the adequacy and validity of the indicators, tools, and systems for monitoring, measuring and verifying results	*Suggestion 1*	*Fully attended*	*Mentions the topic*	
		*Partially attended *	*Mentions the topic associating it positively to the implementation and/or results *	
		*Not attended *	*Mentions the topic associating it with implementation difficulties*	
		*Not applicable*	*Does not mention the topic*	
	*Suggestion 2*	*Fully attended*	*Mentions the topic*	
		*Partially attended *	*Mentions the topic associating it positively to the implementation and/or results *	
		*Not attended *	*Mentions the topic associating it with implementation difficulties*	
		*Not applicable*	*Does not mention the topic*	
	*Suggestion (…)*			
Assess the adequacy of financial, human and material resources to meet the expected results and planning evaluation theoretical background and design and their alignment to the target region context	*Suggestion 1*	*Fully attended*	*Mentions the topic*	
		*Partially attended *	*Mentions the topic associating it positively to the implementation and/or results *	
		*Not attended *	*Mentions the topic associating it with implementation difficulties*	
		*Not applicable*	*Does not mention the topic*	
	*Suggestion 2*	*Fully attended*	*Mentions the topic*	
		*Partially attended *	*Mentions the topic associating it positively to the implementation and/or results *	
		*Not attended *	*Mentions the topic associating it with implementation difficulties*	
		*Not applicable*	*Does not mention the topic*	
	*Suggestion (…)*			

For each evaluation suggestion, one of the options in the third column and one of the options in the fourth column should be ticked

Legend: Evaluability Parameters/dimensions – correspond to the 3 dimensions of analysis considered in the evaluability study. Suggestions - suggestions for improving the intervention plan formulated in the evaluability study. Translation of the recommendation into the intervention plan – The evaluability suggestion was attended (final version of the plan)? Evaluation report mentions the issue that led to the recommendation – The evaluator mention the issue that led to the recommendations? If not, can this be attributed to the change made to the plan during the evaluability study? Comment on impact of the recommendation (how the subject is reflected in the report) – After reading the report in the light of the evaluability recommendations and filling in the previous columns, the meta-evaluator should produce a brief qualitative judgement on the impact of the recommendation on the intervention and its results
